# Twitter: A Good Place to Detect Health Conditions

**DOI:** 10.1371/journal.pone.0086191

**Published:** 2014-01-29

**Authors:** Víctor M. Prieto, Sérgio Matos, Manuel Álvarez, Fidel Cacheda, José Luís Oliveira

**Affiliations:** 1 Department of Information and Communication Technologies, Campus Elviña s/n, Coruña, Spain; 2 University of Aveiro, DETI/IEETA, Campus Universitariá de Santiago, Aveiro, Portugal; University of Oxford, United Kingdom

## Abstract

With the proliferation of social networks and blogs, the Internet is increasingly being used to disseminate personal health information rather than just as a source of information. In this paper we exploit the wealth of user-generated data, available through the micro-blogging service Twitter, to estimate and track the incidence of health conditions in society. The method is based on two stages: we start by extracting possibly relevant tweets using a set of specially crafted regular expressions, and then classify these initial messages using machine learning methods. Furthermore, we selected relevant features to improve the results and the execution times. To test the method, we considered four health states or conditions, namely flu, depression, pregnancy and eating disorders, and two locations, Portugal and Spain.

We present the results obtained and demonstrate that the detection results and the performance of the method are improved after feature selection. The results are promising, with areas under the receiver operating characteristic curve between 0.7 and 0.9, and f-measure values around 0.8 and 0.9. This fact indicates that such approach provides a feasible solution for measuring and tracking the evolution of health states within the society.

## Introduction

The Internet constitutes a huge source of information. Since it became a widespread service, it has been used by individuals seeking medical information. However, with the advent of the Web2.0 paradigm, the Internet is now used not only as an information source but also to disseminate personal health information, experiences and knowledge [1], [2].

Much of this health related information is shared through social media platforms such as Twitter and Facebook. Twitter (http://www.twitter.com), for example, offers a micro-blogging platform that allows users to communicate through status updates limited to 140 characters, commonly referred to as “tweets”. It has over 200 million active users (https://twitter.com/twitter/status/281051652235087872), and around 400 million tweets are published daily. These large quantities of user generated content (UGC) can be used in different ways, representing great opportunities for data and text mining approaches in many fields of application. Mining these data provides an instantaneous snapshot of the public's opinions, and longitudinal tracking allows identification of changes in opinions [3]. This applies also to health related information, as can be verified by the various works that use Twitter and other user-generated data to assess and categorize the kind of information sought by individuals, to infer health status or measure the spread of a disease in a population. In a previous work by Lyon et al., for example, the authors compared three web-based biosecurity intelligence systems and highlighted the value of social media, namely Twitter, in terms of the speed the information is passed and also because many issues or messages were not disseminated through other means [4]. The greatest advantage of these methods over traditional ones is instant feedback: while health reports are published in a weekly or monthly basis, UGC such as tweets and search engine query logs can be obtained and processed almost instantly. This characteristic is of extreme importance because early stage detection can reduce the impact of epidemic breakouts [5], [6].

In this work, we propose an automated method, taking advantage of the wealth of data provided by Twitter, to measure the incidence of a set of health conditions in society. The method, detailed in the following sections, is based on two main steps: first, tweets are filtered using specially crafted regular expressions; second, filtered tweets are manually labelled as positive or negative and are used to train classifiers. Once accurate filters and classifiers are created, these can be applied to incoming streams of Twitter data allowing measuring and tracking the incidence of health conditions. We focused our work on two official languages in the Iberian peninsula (Portuguese and Spanish) and in the analysis of four health states, namely flu, depression, pregnancy and eating disorders. We acquired more than 10 million tweets and performed a set of stages in order to obtain the final filtering and classification strategy. The proposed method is general and may be automatically applied (e.g. South America) or adapted for other regions and health conditions. Furthermore, each step of our method was implemented using freely available tools and is described in detail, facilitating the reproduction of the research work described in this paper [7].

The structure of this article is as follows. In Section 2 we comment on related works regarding the retrieval of health information from social media. In Section 3 we describe the proposed method, explaining the details of each of the method's stages, the datasets used and the experiments performed. In Section 4 we present the results and the improvements obtained in terms of efficacy and performance when using feature selection. Finally, Section 5 includes our conclusions and the possible future work in this field.

## Related Work

Several works regarding the retrieval of health information from social media have already been published, with a major focus on measuring the incidence rate of influenza. In an early work, Chew and Eysenbach suggested a complementary infoveillance approach based on Twitter, and applied it to data obtained during the 2009 H1N1 pandemic. They performed content and sentiment analysis on 2 million tweets containing the keywords “swine flu”, “swineflu”, or “H1N1”. For this, they created a range of queries related to different content categories, and showed that the results of these queries correlated well with the results of manual coding, suggesting that near real-time content and sentiment analysis could be achieved, allowing monitoring large amounts of textual data over time [3]. In another work, Signorini et al. collected tweets matching a set of 15 pre-specified search terms including “flu”, “vaccine”, “tamiflu”, and “h1n1” and applied content analysis and regression models to measure and monitor public concern and levels of disease during the H1N1 pandemic in the United States. Using a regression model trained on 1 million influenza-related tweets and using the incidence rates reported by the Centers for Disease Control (CDC) as reference, the authors reported errors ranging from 0.04% to 0.93% for the estimation of influenza-like illness levels [8]. Regression models were also used to estimate flu incidence rates in the United Kingdom [9] and in the United States [10], [11], leading to correlation coefficients of approximately 0.95 for the linear regression between the estimates obtained and flu statistics reported by the official entities in each country. Aramaki et al. applied SVM machine learning techniques to Twitter messages to predict influenza rates in Japan, achieving a Pearson's correlation ratio of 0.89 against the officially reported incidence rates [5]. In a recent work, Santos and Matos combined data from Twitter and search engine logs in a regression model to estimate the incidence of flu in Portugal, achieving a Pearson's correlation ratio of 0.89 against the results of incidence statistics estimated by the Influenzanet project (https://www.influenzanet.eu/) [12]. Chunara et al. analysed cholera-related tweets published during the first 100 days of the 2010 Haitian cholera outbreak. For this, all tweets published in this period and containing the word “cholera” or the hashtag “#cholera” were considered, and these data were compared to data from two sources: HealthMap, an automated surveillance platform, and the Haitian Ministry of Public Health (MSPP). They showed good correlation between Twitter and HealthMap data, and showed a good correlation (0.83) between Twitter and MSPP data in the initial period of the outbreak, although this value decreased to 0.25 when the complete 100 days period was considered [13].

Apart from analysing the incidence of flu and infectious diseases related events, the analysis of other health parameters using Twitter data has also been reported. Scanfeld et al., for example, applied content analysis to 1000 tweets to explore evidence of misunderstanding or misuse of antibiotics [2]. Heaivilin et al. also applied content analysis to a set of 1000 tweets matching search criteria relating to dental pain. The content was coded using pre-established categories, including the experience of dental pain, actions taken or contemplated in response to a toothache, impact on daily life, and advice sought from the Twitter community [14]. Bosley et al. analysed and categorized 60 thousand tweets concerning cardiac arrest and resuscitation, obtained during a 38 day period using a set of seven search terms [15]. These works demonstrate that Twitter data can be used to study a broad range of health related issues. In this work we combined a content analysis step with a classification step in order to identify positive mentions about the incidence of disease and other health conditions, and evaluated the combined approach on data in two languages and for four distinct conditions.

## Methods

In this article we propose a method to extract a set of tweets that show the presence of certain health conditions in people, as a way to infer the incidence of such conditions in society. This section describes the procedures used to obtain the tweets related to these conditions.

To acquire the tweets for this study, we developed an application that uses the Twitter search API [16] and the geocoding information contained in the tweet metadata to obtain only tweets originated in Spain and Portugal. Furthermore, in order to filter out tweets not written in Spanish or Portuguese, we used a freely available language detection library [17]. This library is based on Bayesian filters and has a precision of 0.99 in detecting the 53 languages it supports. Tweets were acquired during 30 days (from October 30th to November 30th, 2012), producing datasets with approximately 5.8 and 4.5 million tweets for Spanish and Portuguese, respectively.

After obtaining the datasets, the following stages were performed in order to obtain the final filtering and classification strategy:

- Define several regular expressions to extract only the tweets related to the studied diseases. The method can be applied to another group of diseases, requiring the creation of a different set of regular expressions.
- In order to filter those tweets that match with the regular expressions but that do not indicate the presence or absence of a disease in one person, we apply machine learning. For this, we tagged a set of tweets as being positive or negative for each health condition, and defined a set of features to be used to train the classifiers.
- Finally, since a large feature set is generated, we use feature selection in order to improve the results, the computational cost and the execution time.

These steps are explained in detail in the following sections.

### 3.1 Regular expressions

To create these expressions, we initially obtained a set of tweets containing the name of each condition, removing tweet replies (re-tweets) and tweets that included links, and calculated the log-likelihood of the words that occurred within those datasets, obtaining an ordered list of words associated with each condition. Based on these lists, on manual content analysis and on general knowledge about the studied health states, we then defined the regular expressions for each specific case.

Tables 1 and 2 show the regular expressions used to detect tweets related to the analysed health conditions in Spanish and Portuguese, respectively. These are described here:

**Table 1:** Regular expressions for detecting health disorders in Spanish tweets.

Flu	Regular Expresion
Flu	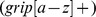
Cold	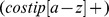
	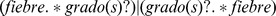
Flu Symptoms	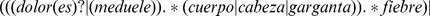
	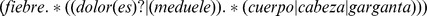
**Pregnancy**	
Pregnancy	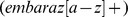
Common phrases	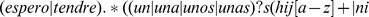 *Ã±* 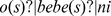 *Ã±* 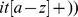
	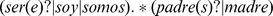
**Depression**	
Depression	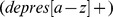
Depressed	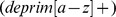
Common phrases	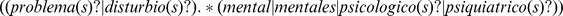
	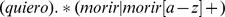
	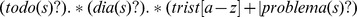
**Eating Disorders**	
Obesity	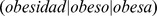
Overweight	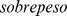
Bulimia	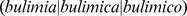
Anorexia	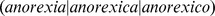
Bigorexia	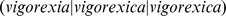
Ebigorexia	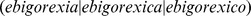
Orthorexia	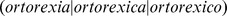
Common phrases	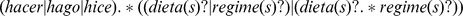
	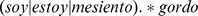
	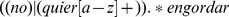
	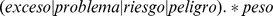
	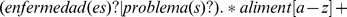

**Table 2:** Regular expressions for detecting health disorders in Portuguese tweets.

Flu	Regular Expresion
Flu	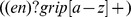
Cold	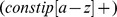
Flu Symptoms	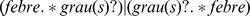
	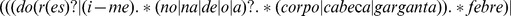
	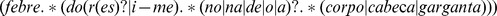
**Pregnancy**	
Pregnancy	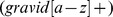
Common phrases	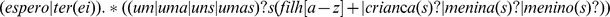
	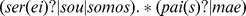
**Depression**	
Depression	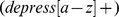
Depressed	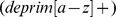
Common phrases	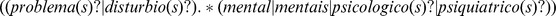
	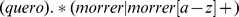
	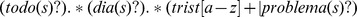
**Eating Disorders**	
Obesity	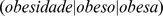
Overweight	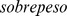
Bulimia	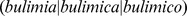
Anorexia	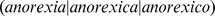
Bigorexia	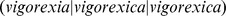
Ebigorexia	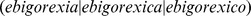
Orthorexia	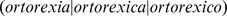
Common phrases	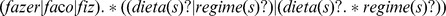
	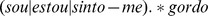
	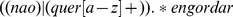
	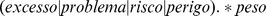
	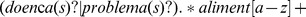

- Flu: regular expressions used are related to flu and its variants, and also to known symptoms of the disease.
- Pregnancy: for obtaining tweets related to pregnancy we used different variations of the word based on its lexeme. We also used words and short phrases identified in the manual analysis of the tweets, as for example, the words used to celebrate the birth of a child.
- Depression: we used words and symptoms related to depression.
- Eating disorders: we used the names of different eating disorders, and various phrases and words that are usually related to these. We have considered the following eating disorders: anorexia, bulimia, bigorexia, obesity and orthorexia.

Table 3 shows the number of tweets considered for each language and disease pair, after applying the regular expressions shown in Tables 1 and 2.

**Table 3:** Number of examples and features in each dataset.

	Spanish		Portuguese	
	Tweets	Features	Tweets	Features
Depression	3253	721	2846	983
Pregnancy	1985	698	2629	1042
Flu	827	608	1153	842
Eating Disorders	412	567	468	747

### 3.2 Machine learning

The use of regular expressions allowed obtaining large sets of tweets related to the specified diseases. However, among the obtained tweets, negative sentences that do not indicate the presence or absence of a disease in one person, such as “Hoping the flu does strike me again this winter”, may also occur. To solve this problem, we applied machine learning techniques on the datasets obtained using the regular expressions, in order to filter such cases. This allowed differentiating the tweets that only mention a given disease from those which actually indicate that the person has the disease.

In order to apply machine learning, each tweet has to be represented by a set of features. We used a simple bag-of-words (BOW) model after removal of stopwords (from http://snowball.tartarus.org/) and word stemming [18]. We also included character bigrams as extra features, since previous results showed that these features could be helpful in classifying the tweets, as opposed to word bigrams, which did not seem to contain any additional discriminating information. According to the language and the condition studied, we obtained different sets of features, as shown on Table 3.

In order to identify the best classifier for our method, we tested various machine learning techniques (Support Vector Machines (SVM), Naïve Bayes (NB), Decision Trees (DT) and Nearest Neighbour (kNN)) using the obtained features. To test these techniques, we used WEKA [19], a free software tool for data mining and machine learning that includes multiple implementations of different existing techniques. We used 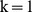 for the kNN classifier, and kept the default parameters in WEKA for each of the machine learning algorithms.

#### 3.2.1 Manual dataset annotation

Before executing the experiments we tagged the different sets of tweets. Each tweet was considered positive, negative or undecided (ambiguous) if:

- Positive: the tweet content indicates the presence of one of the studied diseases/states in the person who has written the tweet.
- Negative: the tweet content indicates that the person who wrote it does not have any of the diseases/states studied.
- Undecided: the tweet does not fit into any of the above criteria.

The tagging process was performed by two medical doctors from Portugal and three computer engineers from Spain. A simple computer program was created that iteratively showed a new tweet to the annotator, who read the text shown on the computer screen and pressed a key in the keyboard according to the desired classification, allowing the task to be performed in a very efficient manner. The task was performed within a window of fifteen days but in fact, although this was not measured, the total amount of time taken to annotate the whole dataset was much smaller. Still, there is always some amount of manpower required for creating these types of datasets. Therefore, for larger scale experiments this manual annotation step should serve as the initial input for self-training approaches.

In the Portuguese data set, we only considered those tweets where agreement between the annotators was achieved, and the remaining were considered as undecided. For the Spanish data set a majority voting scheme was used, and tweets were tagged as undecided if no majority existed. For the classification process the tweets tagged as undecided were removed from the dataset, that is, a tweet was only used for the classification if it was labelled as positive or negative. Table 4 shows the number of tweets labelled as positive and negative for each language and health condition.

**Table 4:** Number of tweets labelled as positive, negative and undecided.

	Spanish Tweets			Portuguese Tweets		
Disease	Positive	Negative	Undecided	Positive	Negative	Undecided
Depression	160	3093	0	120	2725	1
Pregnancy	65	1920	0	38	2588	3
Flu	663	164	0	649	501	3
Eating Disorders	111	301	0	87	368	13

### 3.3 Improving the feature sets

Bag-of-words and character n-gram features usually contain several redundant or irrelevant features. So, in this step a subset of relevant features is selected and used for creating the classification model.

The feature selection process provides the following advantages:

- It removes noisy or redundant features. This noise makes it more difficult to discover meaningful patterns.
- To discover good classification patterns, larger training datasets are required. However, in several data mining applications the training dataset is very small. In this case, the models based on a smaller set of features, obtained by applying feature selection, can be useful.
- It improves the efficacy of the classifiers.
- It reduces the training and executing times.

There are two types of approaches for performing feature selection, described as wrapper and filter methods [20]–[22]. The wrapper method uses the intended learning algorithm itself to evaluate the usefulness of the features. On the other hand, the filter method analyses the features according to heuristics based on general characteristics of the data. Usually, the wrapper approach obtains better feature subsets but its computational cost and execution time is higher than the filter approach. For this reason, we only considered filter methods for this work.

The feature selection process has two components: feature evaluator, the algorithm that determines the quality of the subset of features to discriminate the class label; and search method, the method used to search relevant features subsets. Since this problem grows with the number of features, there are several strategies to search the relevant subsets in an efficient way.

The following feature selection algorithms were tested:

- Correlation-based feature selection (CFS): evaluates the relevance of a subset of attributes by considering the individual predictive capability of each feature, and the degree of redundancy between them. So, the subsets preferred are those that are highly correlated with the class label and the correlation between them is low. More details about this algorithm can be found in previous work [23].
- Pearson correlation: this algorithm determines the relevance of a feature by measuring the Pearson correlation between it and the class.
- Gain Ratio: evaluates the worth of an attribute by measuring the gain ratio with respect to the class. The gain ratio of an attribute is obtained through the following equation, where 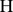 represents the entropy function.
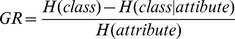

- Relief: evaluates the worth of an attribute by repeatedly sampling an instance and considering the value of the particular attribute for the nearest instance of the same and different class. That is, Relief evaluates the attributes according to how their values distinguish among instances that are near each other.

Detailed descriptions of the Gain Ratio and Relief methods for feature selection can be found in the literature [24]–[26].

## Results

In this section, we present the results obtained with the proposed method to measure the incidence of different diseases, and compare the performance of the different classifiers tested. As can be seen from Table 4, the data are in most cases extremely unbalanced, which makes the choice of the evaluation metric very important. For example, a majority classifier would yield very high accuracy in most of the cases considered, but since the majority class is the negative one, this would amount to a recall (or sensitivity) of zero. Therefore, for the analysis of the results, we considered as the main metric the area under the receiver operating characteristic (ROC) curve (AUC), as it is a robust measure for comparing different classifiers [27]. Additionally, we also present results for the common performance measures of precision, recall and F-measure:

- Precision: the proportion of cases classified as positive by an algorithm, which are actually positive;
- Recall: the proportion of positive cases that were correctly classified as positive;
- F-measure: the harmonic mean of precision and recall, that is, 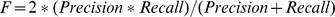;
- Area under the ROC curve (AUC): The ROC curve represents the rate of true positives versus false positives, at various threshold settings. The area under the curve gives an indication of the discriminatory value of the classifier at different operating points.

### 4.1 Results without feature selection

We tested four classifiers, using all the features for each health state and each country: Naïve Bayes, SVM, Decision Trees and kNN. Table 5 shows the results of each type of classifier on the health conditions studied for Spanish and Portuguese, based on 10-fold cross validation. Results varied between 0.6 and 0.9 AUC, with Naïve Bayes classifiers achieving the best overall performance. A large variability in the results is observable, with the best results obtained in the classification of ‘depression’, in both languages, and ‘pregnancy’ in Portuguese tweets. On the other hand, the lowest results were obtained in the classification of tweets related to ‘eating disorders’, with AUC between 0.6 and 0.7 in most cases. Regarding the country, better results were in general obtained in the Portuguese dataset. In terms of F-Measure, most classifiers achieved results between 0.7 and 0.9, with Decision Trees producing the best results in almost all the cases.

**Table 5:** Results obtained on the datasets.

		Spanish Tweets				Portuguese Tweets			
Disease	Classifier	F-Measure	Precision	Recall	AUC	F-Measure	Precision	Recall	AUC
Depression	Naïve Bayes	0.913	0.949	0.891	0.878	0.912	0.947	0.887	0.833
	SVM	0.946	0.948	0.944	0.739	0.902	0.934	0.876	0.691
	Decision Tree	0.976	0.968	0.985	0.845	0.974	0.963	0.985	0.762
	kNN	0.862	0.937	0.814	0.784	0.900	0.937	0.871	0.768
Pregnancy	Naïve Bayes	0.952	0.948	0.957	0.703	0.977	0.973	0.982	0.877
	SVM	0.940	0.942	0.939	0.644	0.945	0.975	0.920	0.679
	Decision Tree	0.947	0.944	0.951	0.689	0.978	0.971	0.985	0.801
	kNN	0.949	0.945	0.953	0.701	0.979	0.975	0.985	0.714
Flu	Naïve Bayes	0.766	0.759	0.775	0.743	0.667	0.667	0.669	0.746
	SVM	0.755	0.749	0.764	0.696	0.681	0.691	0.690	0.671
	Decision Tree	0.779	0.757	0.804	0.670	0.672	0.672	0.674	0.746
	kNN	0.761	0.756	0.799	0.786	0.687	0.687	0.689	0.745
Eating Disorders	Naïve Bayes	0.720	0.720	0.720	0.714	0.786	0.785	0.817	0.744
	SVM	0.683	0.688	0.679	0.607	0.725	0.729	0.720	0.650
	Decision Tree	0.785	0.756	0.817	0.630	0.869	0.838	0.902	0.690
	kNN	0.684	0.714	0.669	0.696	0.667	0.737	0.630	0.686

AUC = Area Under the receiver operating characteristic Curve.

### 4.2 Feature selection

As can be seen from Table 3, the number of cases available to train the classification models are of the same order of magnitude as the number of features extracted. In order to minimize this problem, we estimated the optimal number of features to use for each condition and each language. Table 6 shows the size of the feature subsets obtained with the four feature selection algorithms tested. To obtain these results we iteratively changed the number of features to be considered, and selected, for each combination of condition, language and feature selection method, the smallest subset that allowed achieving similar or better results than those obtained using all the features. The comparison was based on the average of ten cross-validation folds, with the same number of features used in each fold. Comparing with Table 3, we can see that, independently of the language and the cases, both CFS and Pearson correlation selection techniques allowed eliminating over 90% of features. In the case of the gain ratio method, the results are similar except in the case of ‘flu’, where a larger number of features had to be considered, due to the difficulty of this classification. In this case, only around half of the features were filtered. When using the Relief method, the number of features was reduced by only 70% or 80%, in some cases.

**Table 6:** Number of features selected by each selection method.

	Spanish				Portuguese			
	CFS	Pearson	Gain Ratio	Relief	CFS	Pearson	Gain Ratio	Relief
Pregnancy	52	51	51	51	31	51	51	201
Depression	55	51	51	51	62	51	51	201
Flu	74	51	301	101	52	51	301	201
Eating Disorders	58	51	51	51	62	51	51	101

The cross-validation results obtained using the reduced subsets of features are shown in four tables, according to the feature selection algorithm applied. Statistically significant differences (two-tailed t-test, 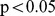) to the use of all features are marked with ‘+’, for positive differences (i.e., the classifier performed better after feature selection), and ‘−’, for negative differences. In these experiments we only show results for Naïve Bayes and kNN because these classifiers obtained the best overall results when considering the AUC metric, both using the complete set of features and after applying the feature selection methods. Additionally, these classifiers are computationally less expensive, which favours their use when extending this method to other cases or languages.

In Table 7, we show the results obtained on the subsets generated using CFS. The results show that Naïve Bayes obtained better results than the ones obtained using all the features. In most cases, Naïve Bayes improved by more than 0.1 for the different analysed metrics. On the other hand, kNN also obtained better results than the ones obtained with all features, except in the Spanish tweets for ‘Flu’, where it obtained slightly worse results.

**Table 7:** Classification results obtained on subsets generated using CFS.

		Spanish Tweets				Portuguese Tweets			
Disease	Classifier	F-Measure	Precision	Recall	AUC	F-Measure	Precision	Recall	AUC
Depression	NB	0.913+	0.949	0.890−	0.892	0.912+	0.949	0.888+	0.861
	kNN	0.932+	0.943	0.923+	0.876+	0.934+	0.931−	0.938+	0.821
Pregnancy	NB	0.955	0.953	0.958	0.832	0.982	0.981	0.985	0.882
	kNN	0.950+	0.942−	0.959+	0.774	0.979+	0.972−	0.985+	0.847+
Flu	NB	0.818	0.813	0.828+	0.818+	0.688	0.687	0.690	0.752
	kNN	0.755−	0.748−	0.796−	0.677−	0.572	0.687	0.690	0.752
Eating Disorders	NB	0.762	0.765	0.760+	0.796	0.844+	0.841+	0.853+	0.858+
	kNN	0.705	0.765+	0.760+	0.796+	0.782+	0.773+	0.800+	0.762+

Statistically significant differences (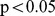), as compared to using all the features and measured by a two-tailed t-test, are marked as ‘+’ (positive differences) and ‘−’ (negative differences).

The results obtained using Pearson correlation are shown in Table 8. We observe that Naïve Bayes and kNN achieved very similar results to those obtained using the complete subsets of features. In some cases, a small improvement was obtained while in others there was a small decline of the results. However, these results were obtained with a much lower number of features.

**Table 8:** Classification results obtained on subsets generated using Pearson correlation.

		Spanish Tweets				Portuguese Tweets			
Disease	Classifier	F-Measure	Precision	Recall	AUC	F-Measure	Precision	Recall	AUC
Depression	NB	0.902	0.946−	0.875	0.872	0.900−	0.947	0.868−	0.832
	kNN	0.927+	0.941	0.916+	0.840+	0.934+	0.932−	0.934+	0.783
Pregnancy	NB	0.919−	0.955+	0.893−	0.778	0.961	0.981	0.946	0.892
	kNN	0.946−	0.944−	0.949−	0.701	0.974−	0.973−	0.976−	0.872+
Flu	NB	0.815	0.812	0.820	0.788+	0.672	0.672	0.674	0.744
	kNN	0.761+	0.752	0.973+	0.643−	0.584−	0.601−	0.607−	0.628
Eating Disorders	NB	0.759	0.760	0.743	0.781	0.834	0.838	0.831+	0.849+
	kNN	0.714+	0.717	0.711+	0.680	0.721+	0.752+	0.701+	0.685−

Statistically significant differences (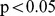), as compared to using all the features and measured by a two-tailed t-test, are marked as ‘+’ (positive differences) and ‘−’ (negative differences).

Table 9 shows the results obtained by Naïve Bayes and kNN on the subsets generated using Gain Ratio. In the case of ‘flu’, we have used a larger number of features (301) than in the previous cases. This is due to the difficulty in classifying ‘flu’ and to the fact that this method did not select the appropriate features.

**Table 9:** Classification results obtained on subsets generated using Gain Ratio.

		Spanish Tweets				Portuguese Tweets			
Disease	Classifier	F-Measure	Precision	Recall	AUC	F-Measure	Precision	Recall	AUC
Depression	NB	0.919+	0.949	0.900+	0.909+	0.944+	0.942−	0.958+	0.864+
	kNN	0.936+	0.932	0.943+	0.844+	0.937+	0.926−	0.957+	0.813
Pregnancy	NB	0.954	0.949+	0.963	0.750	0.979	0.980	0.971	0.879
	kNN	0.951−	0.936−	0.967+	0.743	0.978−	0.972−	0.984−	0.849+
Flu	NB	0.803+	0.800	0.808+	0.797	0.719	0.713	0.714	0.786+
	kNN	0.773	0.770	0.805+	0.690−	0.736+	0.736+	0.736+	0.673−
Eating Disorders	NB	0.710−	0.797+	0.767+	0.729+	0.786+	0.800+	0.826+	0.750+
	kNN	0.640−	0.739+	0.735+	0.710+	0.738+	0.850+	0.815+	0.731+

Statistically significant differences (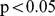), as compared to using all the features and measured by a two-tailed t-test, are marked as ‘+’ (positive differences) and ‘−’ (negative differences).

The results achieved by Relief are shown in Table 10. In this case to achieve similar results of the ones obtained using all features, we have used more features that in the previous cases (CFS, Pearson, Gain Ratio). In the Portuguese subsets, the number of features is almost four times more than the ones selected by Pearson correlation, for example. Again, although the results did not improve significantly, we have achieved similar results to the obtained using all features, with a smaller number of features.

**Table 10:** Classification results obtained on subsets generated using Relief.

		Spanish Tweets				Portuguese Tweets			
Disease	Classifier	F-Measure	Precision	Recall	AUC	F-Measure	Precision	Recall	AUC
Depression	NB	0.904	0.946−	0.877	0.873	0.885−	0.945	0.842−	0.820
	kNN	0.924+	0.942	0.911+	0.820+	0.925+	0.936	0.915+	0.787
Pregnancy	NB	0.893−	0.950+	0.849−	0.711	0.961	0.981	0.945	0.887
	kNN	0.945−	0.943−	0.947−	0.707	0.965−	0.975	0.956−	0.747
Flu	NB	0.785+	0.770	0.748−	0.746+	0.643	0.635	0.635	0.730−
	kNN	0.758	0.747	0.787	0.700	0.670−	0.650−	0.640−	0.695−
Eating Disorders	NB	0.731	0.733	0.728	0.736	0.824	0.826+	0.822+	0.777
	kNN	0.711+	0.707−	0.715+	0.700+	0.781+	0.778+	0.782+	0.677−

Statistically significant differences (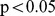), as compared to using all the features and measured by a two-tailed t-test, are marked as ‘+’ (positive differences) and ‘−’ (negative differences).

**Figure 1:**
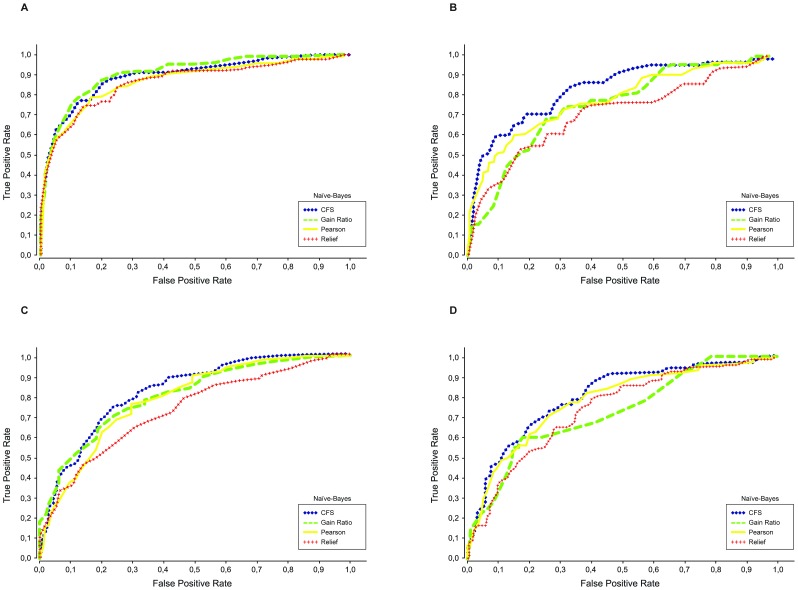
Classifier ROC curves for the Spanish datasets. The ROC curves illustrate the performance for classifying tweets as related to (positive) or not related to (negative) a given health state. Results shown are for a Naïve Bayes classifier trained with the subsets of features generated by four different feature selection algorithms. A) Depression, B) Pregnancy, C) Flu and D) Eating Disorders.

**Figure 2:**
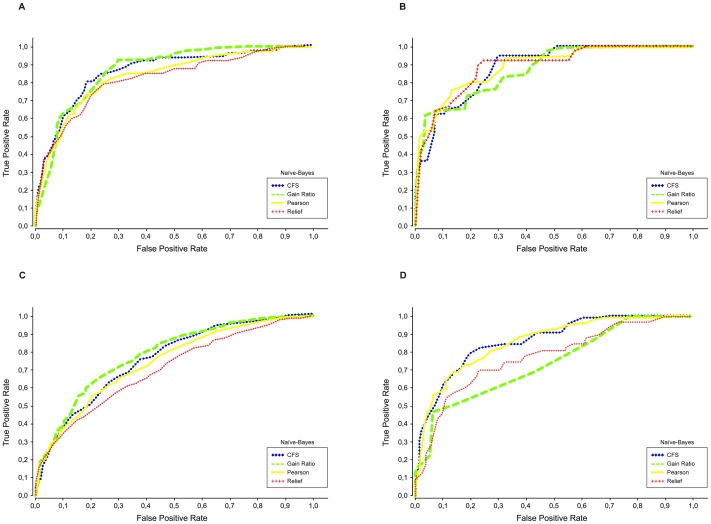
Classifier ROC curves for the Portuguese datasets. The ROC curves illustrate the performance for classifying tweets as related to (positive) or not related to (negative) a given health state. Results shown are for a Naïve Bayes classifier trained with the subsets of features generated by four different feature selection algorithms. A) Depression, B) Pregnancy, C) Flu and D) Eating Disorders.

The ROC curves for the Naïve Bayes classifiers, considering each feature selection method, are shown in Figures 1 and 2, for Spanish and Portuguese tweets respectively. From these curves, we can see that the CFS method often leads to better classifiers. The curves for the kNN classifier show similar characteristics, with the CFS method again producing more consistent results.

In summary, the best results were obtained using Naïve Bayes on the subsets of features generated by CFS. Comparing to the results obtained with the complete feature set (Table 5), these were improved in all the cases, with final AUC and F-measure values between 0.8 and 0.9 in almost all the cases.

There were significant gains in the cases that had the lowest initial results, apart from ‘flu’ in the Portuguese dataset. Specifically, gains in AUC between 0.07 and 0.13 were obtained in the Spanish datasets for ‘flu’, ‘eating disorders’ and ‘pregnancy’, corresponding to percentage improvements between 10% and 18%. In the Portuguese datasets, the best improvement was obtained for the classification of ‘eating disorders’ tweets, with a absolute gain of 0.11 corresponding to a 15% improvement.

Finally, we can say that the proposed method presents promising results and that the feature selection step had, as expected, a very significant impact on these results.

### 4.3 Efficiency results

In the previous section we discussed and demonstrated that using feature selection we can improve the efficacy of the classifiers. In this section we analyse the profit obtained in terms of performance. For that, we analysed the time spent in the generation of the model, and the time used to classify the dataset. The results obtained are shown in Table 11.

**Table 11:** Time (s) spent training the models and performing classification.

Features	Classifier	Training	Classification
All Features	Naïve Bayes	23.25	219.32
	kNN	-	60.95
CFS	Naïve Bayes	0.01	0.01
	kNN	-	15.95
Pearson correlation	Naïve Bayes	0.01	0.10
	kNN	-	15.78
Gain Ratio	Naïve Bayes	0.02	0.22
	kNN	-	20.88
Relief	Naïve Bayes	0.01	0.33
	kNN	-	40.62

The analysis of the results shows that the time spent to generate the model was reduced from over 20 seconds to 0.02 seconds or less. Note that, in the case of kNN, the model is generated during the classification process, and so no training time is considered. Additionally, the time used to make the classification was reduced from around 220 seconds to 40 seconds in the worst case. In short, the time spent to generate the model and to make the classification were reduced by around 99% and 81%, respectively.

## Conclusions

This article presents the results of applying a method based on regular expressions and machine-learning classifiers to identify a set of tweets that show the presence of certain diseases or health states in the society. The proposed method is composed of the following steps: 1) collect an initial set of tweets that mention the health condition to be measured, using known names and synonyms; 2) use log-likelihood measures and manual content analysis to create regular expressions; 3) filter tweets using these regular expressions; 4) label the selected tweets as positive or negative; 5) extract n-gram features and apply correlation-based feature selection (CFS); 6) train (and evaluate) a classifier based on these data. In addition, the regular expressions and classifiers created can be applied to an incoming stream of Twitter data to identify tweets about specific health issues, allowing measuring and tracking the incidence of those health conditions almost in real-time. This approach is general and could in principle be easily adapted to other languages or geographical areas and to other health or social conditions, by creating new regular expressions following the same procedure and by training new classification models.

The method was evaluated in a study centred in Spain and Portugal, based on the geocoded data and on the language of the tweets, and in four conditions: flu, depression, pregnancy, and eating disorders. To the best of our knowledge, there are no studies showing and comparing the application of similar techniques to data in two languages and covering different countries.

We obtained classification results between 0.7 and 0.9 in AUC and also in terms of F-measure. The feature selection algorithms used (CFS, Pearson correlation, Gain Ratio and Relief) reduced the number of features by up to 90%, while at the same time improving the classification results by up to 18% in AUC and up to 7% in F-measure. Additionally, this reduced the time taken to generate the model and to make the classification by around 99% and 81%, respectively. The results obtained over these different health issues and languages indicate that the methodology can generalize to other cases and could be effectively and efficiently used for measuring and tracking the evolution of many health related aspects within the society.

A possible limitation of this approach regarding its application to other languages and/or health conditions is the need for manually annotated training sets. Although this can be done efficiently, the possibility of using self-training techniques should be investigated, in order to reduce the amount of manpower required. Another possible way of improving and extending the method is by including other types of user-generated content, such as Internet searches or comments to news articles, which may also contain information related to some of these aspects. Thus, this data could be used to complement the data extracted from Twitter. Another interesting aspect would be to study the symptoms that appear in tweets related to each of the diseases discussed and compare these to the known symptoms today.
